# Tissue explants as tools for studying the epigenetic modulation of the GH-IGF-I axis in farmed fish

**DOI:** 10.3389/fphys.2024.1410660

**Published:** 2024-06-20

**Authors:** Erick Perera, Javier Román-Padilla, Juan Antonio Hidalgo-Pérez, Rubén Huesa-Cerdán, Manuel Yúfera, Juan Miguel Mancera, Juan Antonio Martos-Sitcha, Gonzalo Martínez-Rodríguez, Juan Bosco Ortiz-Delgado, Carmen Navarro-Guillén, Javier A. Rodriguez-Casariego

**Affiliations:** ^1^ Department of Marine Biology and Aquaculture, Instituto de Ciencias Marinas de Andalucía (ICMAN-CSIC), Spanish National Research Council (CSIC), Puerto Real, Spain; ^2^ Department of Biology, Faculty of Marine and Environmental Sciences, Instituto Universitario de Investigación Marina (INMAR), University of Cadiz, Campus de Excelencia Internacional del Mar (CEIMAR), Puerto Real, Spain; ^3^ Environmental Epigenetics Laboratory, Institute of Environment, Florida International University, Miami, FL, United States

**Keywords:** DNA methylation, epigenetics, fish, GH-IGF axis, *in vitro* culture, tissue explant

## Abstract

Somatic growth in vertebrates is mainly controlled by the growth hormone (GH)/insulin-like growth factor I (IGF-I) axis. The role of epigenetic mechanisms in regulating this axis in fish is far from being understood. This work aimed to optimize and evaluate the use of short-term culture of pituitary and liver explants from a farmed fish, the gilthead seabream *Sparus aurata*, for studying epigenetic mechanisms involved in GH/IGF-I axis regulation. Our results on viability, structure, proliferation, and functionality of explants support their use in short-term assays. Pituitary explants showed no variation in *gh* expression after exposure to the DNA methylation inhibitor decitabine (5-Aza-2′-deoxycytidine; DAC), despite responding to DAC by changing *dnmt3bb* and *tet1* expression, and TET activity, producing an increase in overall DNA hydroxymethylation. Conversely, in liver explants, DAC had no effects on *dnmt*_*s*_ and *tet*_*s*_ expression or activity, but modified the expression of genes from the GH-IGF-I axis. In particular, the expression of *igfbp2a* was increased and that of *igfbp4*, *ghri* and *ghrii* was decreased by DAC as well as by genistein, which is suggestive of impaired growth. While incubation of liver explants with S-adenosylmethionine (SAM) produced no clear effects, it is proposed that nutrients must ensure the methylation milieu within the liver in the fish to sustain proper growth, which need further *in vivo* verification. Pituitary and liver explants from *S. aurata* can be further used as described herein for the screening of inhibitors or activators of epigenetic regulators, as well as for assessing epigenetic mechanisms behind GH-IGF-I variation in farmed fish.

## Introduction

Growth rate in farmed fish is among the phenotypic traits of major relevance for the aquaculture industry. Therefore, several strategies have been followed to increase fish growth rate such as genetic selection (Janssen et al., 2018) and improvements in diet formulation and feeding practices (Glencross et al., 2023). Somatic growth in fish, as in other vertebrates, is mainly controlled by the growth hormone (GH) - insulin-like growth factor I (IGF-I) axis. The molecular and physiological features of this axis in fish have been thoroughly studied and reviewed (Pérez-Sánchez et al., 2018; Bertucci et al., 2019; Triantaphyllopoulos et al., 2020; Ndandala et al., 2022). Briefly, GH is secreted by the pituitary under the control of the hypothalamus, and exerts systemic functions in metabolism, reproduction, physical activity, immunity and osmotic regulation (Vélez and Unniappan, 2021). Regarding growth, the main target for GH action is the liver, where it is recognized by GH receptors and induces the expression and release of IGF-I, which ultimately promote somatic growth. Indeed, circulating IGF-I stands as the most reliable biomarker of growth in fish (Pérez-Sánchez et al., 2018). However, secretion, half live, and actions of IGF-I are dependent on a suit of IGF binding proteins (IGFBPs) (Garcia de la Serrana and Macqueen, 2018). While some paralogs of IGFBPs are more clearly related with growth inhibition in fish, the understanding of the functions of these genes remains fragmented across fish (Garcia de la Serrana and Macqueen, 2018).

Main environmental factors regulating this system are those influencing the nutritional status (Pérez-Sánchez et al., 2018; Bertucci et al., 2019). Given that environmental factors modulate the activity of GH-IGF-I axis in fish, it is plausible that epigenetic mechanisms are involved, as epigenetic modifications serve as links between the environment and the genome to produce adaptive responses. Indeed, it is known that the P2 promoter of IGF-I is epigenetically regulated by DNA methylation in humans, with consequences in circulating IGF-I and growth (Ouni et al., 2016) and that DNA methylation also affects the activity of growth hormone response elements (GHREs) modulating IGF-I expression (Fu et al., 2015). However, the role of epigenetics in regulating GH-IGF-I axis in fish is far from being understood. GH putative promoter methylation in pituitary was observed in females of Nile tilapia compared with males, and it is negatively correlated with mRNA of GH and growth rate (Zhong et al., 2014). In liver, DNA methylation differences in *igf2bp2* are linked to differences in growth in Nile tilapia (Podgorniak et al., 2019) while hydroxymethylation was found in other growth-related genes such as *igfbp2* in this fish species (Konstantinidis et al., 2021). Also, changes in *igf1* gene expression during development are negatively corelated with DNA methylation at promoter and first exon in Japanese flounder (*Paralichthys olivaceus*) (Huang et al., 2018). In the smooth tongue sole (*Cynoglossus semilaevis*), exons in the *igf1* gene had different methylation levels in fish under osmotic stress compared to controls (Li et al., 2017). Results, in addition to sparse, are poorly consistent among fish species.

To further advance the understanding of the epigenetic regulation of the GH-IGF-I axis in farmed fish, and to evaluate environmental manipulations with putative positive effects on growth and other traits, suited experimental models are needed. While studies in live fish are unique to ultimately evaluate growth rate and other physiological responses, *in vitro* models are suited to screening and mechanistic studies. Different cell culture approaches have been used in epigenetic studies, but lack of similarity between the DNA methylation profiles of cultured cell lines and *in vivo* tissues have been reported (Antequera et al., 1990; Nestor et al., 2015). Conversely, it is known that models that better reproduce the architecture of the original tissue produce more realistic responses (Pampaloni et al., 2007), probably because these models enable the cell-cell interactions that occur in the animal. While these models have been developed such as spheroids, organoids, and precision cut liver slices (PCLS), the use of explants is still convenient because they are cheaper and can be implemented in any laboratory having only general lab equipment. Successful short-term explant culture of fish pituitaries (Helms et al., 1987; Astola et al., 2004; Cánepa et al., 2008; Fuentes et al., 2010) and liver (Yu et al., 2008; Beitel et al., 2015; Doering et al., 2015; Eisner et al., 2016; LeClair, 2021) have been reported.

This work aimed to optimize and evaluate the use of short-term culture of pituitary and liver explants from a farmed fish, the gilthead seabream *Sparus aurata*, for studying epigenetic mechanisms and GH/IGF-I axis regulation. We showed that pituitary and liver explants from *S. aurata* can be further used as described herein for the screening of inhibitors or activators of epigenetic regulators, as well as for assessing epigenetic mechanisms behind GH-IGF-I variation in farmed fish.

## Materials and methods

### Fish and housing conditions

Gilthead seabream (*S. aurata*) fish (body weight 37.01 ± 0.83 g; mean ± sem) were obtained from University of Cadiz, and maintained at Institute for Marine Science of Andalusia (ICMAN-CSIC) facilities in aerated 120 L tanks in an open system under natural conditions of photoperiod and temperature (19–22^o^C), salinity of 34 ppt, oxygen level above 90%, pH 8.0 ± 0.1, and NH_4_ < 0.25 mg L^−1^. Animals were fed by hand to satiety twice a day with a commercial diet (Skretting, Burgos, Spain). All procedures complied with the Guidelines of the Spanish (RD53/2013) and European Union Council (2010/63/EU) for the use and experimentation of laboratory animals, and were reviewed and approved by the Spanish National Research Council (CSIC) Bioethical Committee and that of the Autonomous Andalusian Government (reference number 24/10/2023/092).

### Explant culture optimization

Fish were euthanized with overdose of 2-phenoxyethanol (Sigma-Aldrich, Merck, Germany) and decapitated. Pituitaries were collected and led stand for 1 h in 80% (v/v) Leibovitz L15 medium (L-15), pH 7.4, 10% fetal bovine serum (FBS), 10 mM HEPES, 100 IU/mL penicillin, 100 μg/mL of streptomycin, 2,5 ug/mL amphotericin B, 5.5 mM D(+)-glucose, 380 mOsm adjusted with NaCl, and filtered (before antibiotics addition) through Millipore 0.2 µm, henceforth referred to as culture media. Thereafter, glands were randomly distributed in a 96-wells microplate with one gland per well containing 100 µL of culture media. Five glands were taken from fish and immediately stored at −80^o^C as an initial sample. Pituitaries were cultured for 2 days at 23^o^C under 95% O_2_ and 5% CO_2,_ with gently agitation. Culture media was removed after 24 h, stored at −80°C, and replaced by fresh media. At 24 and 48 h, five pituitaries were frozen at −80^o^C for biochemical assays and five glands stored in RNAlater for DNA and RNA extraction.

For liver explants, livers were placed on iced sterile petri dishes, and diced by hand initially into approximately 3–4 mm^3^ cubes. These explants were used to evaluate, during 48 h, the effect of the number of washing steps (two or five; every 10 min) and agitation during incubation in the viability of the explant. Later, after 5 washes, the effect of different volume of media (100 μL, 500 μL, 1 mL) and size of explant (1–2 mm^3^ or 3–4 mm^3^) were evaluated under gently and continuous agitation. A single 3–4 mm^3^ explant was placed in each well, while for 1–2 mm^3^ explants three explants were used per well. Sterile 96-well plates were used for 100 µL incubations and 24-wells plates for all other incubations. Next, the effect of temperature (ambient or 4^o^C) during washing steps of tissue was assessed in 1–2 mm^3^ explants washed five times and cultured in 1 mL of media under constant agitation. In all cases, five explants were taken from fish and immediately stored at −80^o^C as an initial sample. Finally, liver explants were cultured under the best conditions determined as above for 48 h at 23^o^C under 95% O_2_ and 5% CO_2_. Culture media was removed at 24 h, stored at −80°C, and replaced by fresh media. At 24 and 48 h, five liver explants were frozen at −80^o^C for biochemical assays and five explants stored in RNAlater for gene expression analyses.

### Biochemical assays for explant integrity and functionality

Lactate dehydrogenase (LDH) activity was used to assess tissue integrity and measured by the LDH Activity Assay Kit (Sigma-Aldrich, St. Louis, MO, USA) in both explants and culture media. Catalase activity (CAT) was measured in post-mitochondrial supernatant (PMS) of explants as an indicator of oxidative stress during culture, by measuring the decomposition of H_2_O_2_ (Clairborne, 1985). Briefly, samples were homogenized in 50 μL of ultra-pure water, diluted (1:1) in K-phosphate buffer (0.2 M, pH 7.4) and centrifuged for 10 min at 10,000 g (4°C). The PMS was kept in −80°C until analysis. The enzymatic reaction was performed in 96-well microplates by the addition of 10 μL of the PMS to 140 μL of K-phosphate buffer (0.05 M, pH 7.0) and 150 μL of 30% H_2_O_2_. The decomposition of H_2_O_2_ was kinetically followed at 240 nm. To ensure that low level of CAT activity can be taken as indicative of low level of oxidative stress, explants were exposed to 100 µM of H_2_O_2_ (Ransy et al., 2020) for 15 min, and then collected and examined for CAT activity. GH and IGF-I secreted into the media by pituitaries and liver explants, respectively, were measured by the Fish GH and Fish IGF-I ELISA kits (CUSABIO, Wuhan, China). These ELISAs have been used before in different salmonids and non-salmonid fish, including the gilthead seabream (Amri et al., 2020; Rodríguez-Viera et al., 2022). All assays were performed in a microplate reader BioTek ELx808 (BioTek Instrument, Inc), except for catalase activity that was measured at 240 nm in a Synergy H1 microplate reader (Biotek).

### Histology of explants

Two experiments were performed as above and five explants of each tissue were collected at 0 h (native tissue) and 48 h in 4% formalin buffered to pH 7.0 with 0.1 M phosphate buffer for 24 h. Afterwards, samples were embedded in paraffin following a standard protocol to obtain 5–6 μm histological serial sections (Ortiz-Delgado et al., 2008). The Hematoxylin-Eosin technique (Luna, 1968) was used on deparaffinized slides to evaluate potential morphological differences among the treatment groups. The occurrence of cell proliferation was assessed by immunohistochemistry (PCNA staining) as an additional indicator of tissue functionality. A monoclonal antibody (PC10, Santa Cruz Biotechnology Inc., Heidelberg, Germany) against proliferating cell nuclear antigen (anti-PCNA) was utilized for this purpose, according to Piñuela et al. (2004).

### Exposure of explants to SAM, GEN and DAC

S-adenosylmethionine (SAM) is the main donor of methyl groups for DNA methylation, genistein (GEN) is a methyltransferase inhibitor, and decitabine (5-Aza-2′-deoxycytidine; DAC) is a DNA demethylation agent. For expression of GH-IGF-I related genes, explants were processed as above and exposed during 24 h to three concentrations of SAM (100, 200, 300 µM), GEN (5, 10, 20 µM), and DAC (5, 15, 20 µM) for livers, while pituitaries to two concentrations of SAM (200, 300 µM), GEN (10, 20 µM), and DAC (15, 20 µM). In all cases, control explants were exposed to 0.2% of DMSO. Tissues were collected in RNAlater for gene expression analyses.

### Gene expression of GH-IGF-I axis related genes

DNA and RNA were simultaneously extracted from each sample using the NucleoSpin TriPrep Kit (Macherey-Nagel, Düren, Germany), according to the manufacturer’s protocol. RNA concentration was measured with the Qubit 2.0 Fluorometer (Invitrogen, Life Technologies), and its quality assessed in an Agilent 2,100 Bioanalyzer (Agilent Technologies, Santa Clara, CA, USA) using RNA 6000 Nano Kits. Only samples with an RNA Integrity Number (RIN) higher than 8.5 were used. Total RNA (500 ng from liver and 10 ng from pituitaries) was reverse-transcribed using the qScript DNA synthesis kit (Quanta BioSciences). Gene expression was assessed by real-time qPCR for genes involved in the GH-IGF-I axis (Supplementary Table S1). All primers were synthesized by IDT (Integrated DNA Technologies, Leuven, Belgium). While these primer pairs were used previously (Supplementary Table S1, and references therein), assay linearity and amplification efficiency were corroborated under our conditions to be in all cases above 95% except for *rpl27a* (90%), by making 10-fold dilution curves of template starting from 10 ng. For qPCR, each 10 μL reaction contained 0.5 μL of 200 nM of each primer, 5 μL of PerfeCTa SYBR Green FastMix (Quanta Biosciences) and 4 μL of template (10 ng cDNA for liver and 1 ng for pituitaries). Control reactions with water and RNA instead of cDNA were included. qPCR was performed in a CFX Connect (BioRad) and cycling conditions for all genes were 95 °C, 10 min; [95°C, 15 s; 60°C, 30s] x 40 cycles; melting curve from 65°C to 95°C, with increments of 0.5°C, except for *igfbp2b* and *igfbp4* for which 62°C and 58°C were used for annealing, respectively. Melting curves confirmed in all cases a single amplicon and the absence of primer-dimer artifacts. Relative gene expression was calculated by means of the 2^−ΔΔCT^ method (Livak and Schmittgen, 2001). Six genes were evaluated as housekeeping genes (*ef1a*,*18S*, *rps18*, *gapdh2*, *rpl27a*, *actb*) all showing high stability among treatments according to NormFinder v20 (Andersen et al., 2004). The two most stable genes in each tissue (*ef1a* and *rps18* in pituitary; *ef1a* and *rpl27a* in liver) were chosen for normalization.

### Gene expression of DNA methylation remodeling genes

The gene expression of DNA methylation remodeling genes was performed as detailed above for GH-IGF-I axis related genes, in tissue explants exposed to 15 µM DAC. For these genes, new primers (Supplementary Table S1) were designed in Primer 3 so that they span one exon-exon junction in target mRNAs. These genes showed to be expressed at low levels and thus, six points of 1/5 serial dilutions (*dnmt1*, *dnmt3a, dnmt3l, tet1*, *tet2*, *tet3*) or 1/2 serial dilutions (*dnmt3bb*, *dmap1*) starting from 10 ng of cDNA were used to check for assay linearity (R2) and amplification efficiency (E). In all cases efficiencies were above 98% except for *dmap1* which remained with 71%. Cycling conditions for all these genes were 95 °C, 10 min; [95°C, 15 s; 60°C, 30s] x 40 cycles; melting curve from 65°C to 95°C, with increments of 0.5°C.

### DNA methylation, hydroxymethylation, and activity of DNMT and TET enzymes

DNA methylation and hydroxymethylation were assessed using the MethylFlash Global DNA Methylation (5-mC) ELISA Easy Kit and the MethylFlash Global DNA Hydroxymethylation (5-hmC) ELISA Easy Kit (both from Epigentek, Farmingdale, New York), respectively, in tissue explants exposed to 15 µM DAC. For enzyme activity, nuclear proteins were extracted with the EpiQuik Nuclear Extraction Kit (Epigentek), and activities of DNMT and TET enzymes were measured with the EpiQuik DNMT Activity/Inhibition ELISA Easy Kit and the Epigenase 5 mC-Hydroxylase TET Activity/Inhibition Assay Kit (both from Epigentek), respectively. In all cases, the colorimetric variants of kits were used according to the manufacturer protocols.

### Statistical analysis

All data were checked for normality and homogeneity of variance using Kolmogorov-Smirnov and Levene’s tests, respectively, with *p* < 0.05. LDH data during optimization of explant culture and gene expression after exposure to different concentrations of SAM, GEN and DAC were analyzed by two-way ANOVA, while CAT activity was analyzed by one-way ANOVA. When data were compared with initial values the Dunnett’s test was employed. Differences among means were detected by the Tukey’s test (*p* < 0.05). All other variables (paired data) were compared by t-test (*p* < 0.05). The software package GraphPad Prism 9.0 (GraphPad Software, Inc., San Diego, California, US) was used for all tests performed.

## Results

### Explants retained integrity and functionality for 48 h

**FIGURE 1:**
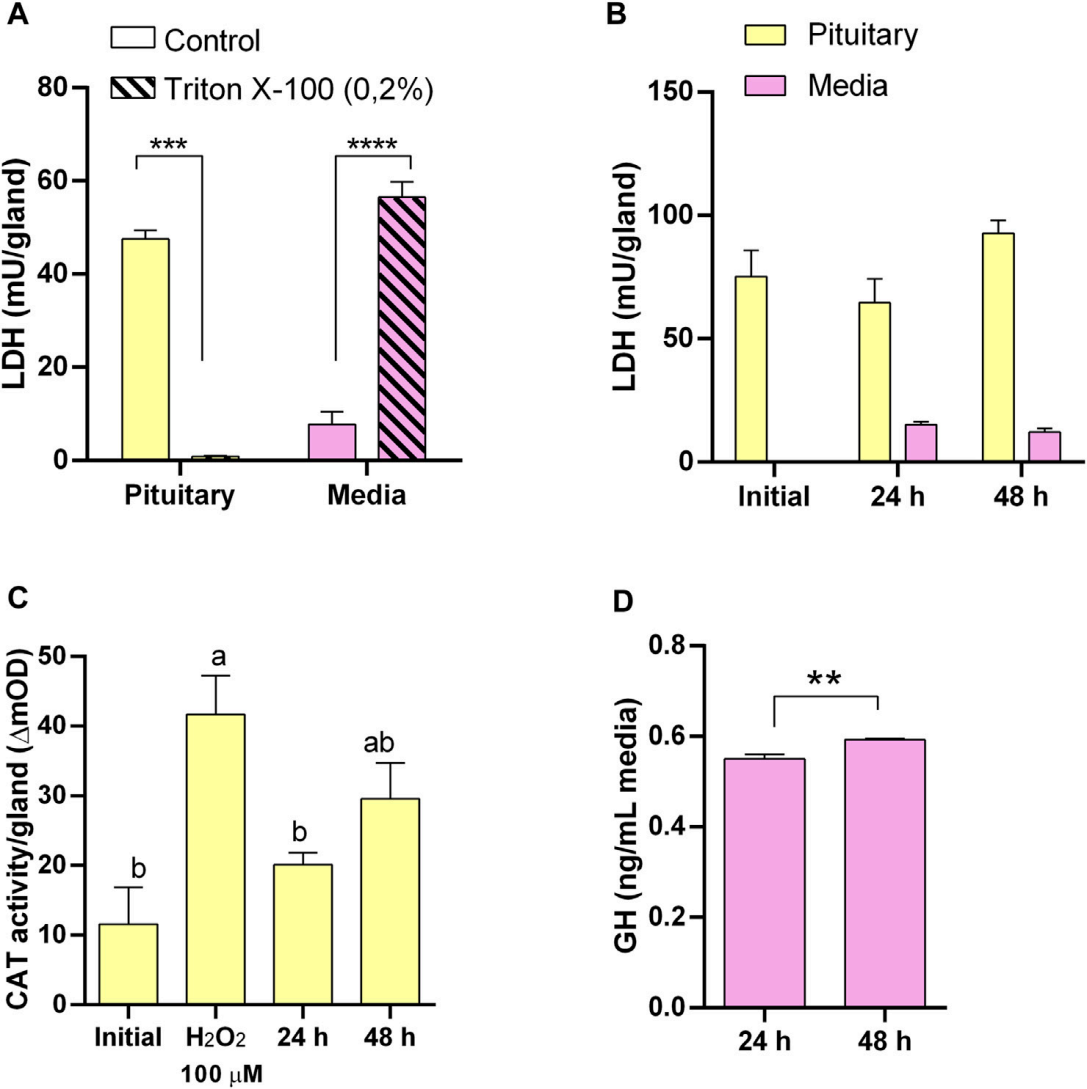
Pituitary explants culture optimization and functionality. **(A)** Lactate dehydrogenase (LDH) activity in pituitary (yellow) and culture media (pink) as suitable indicator of tissue integrity responding to induced damage by 0.2% Triton X-100. **(B)** LDH activity showing pituitary integrity for 48 h in culture. **(C)** Catalase (CAT) activity in pituitary as suitable indicator of oxidative stress responding to induced oxidative stress by H_2_O_2_ and showing low level of oxidative stress in culture. **(D)** Growth hormone (GH) secretion by pituitaries in culture.

**FIGURE 2:**
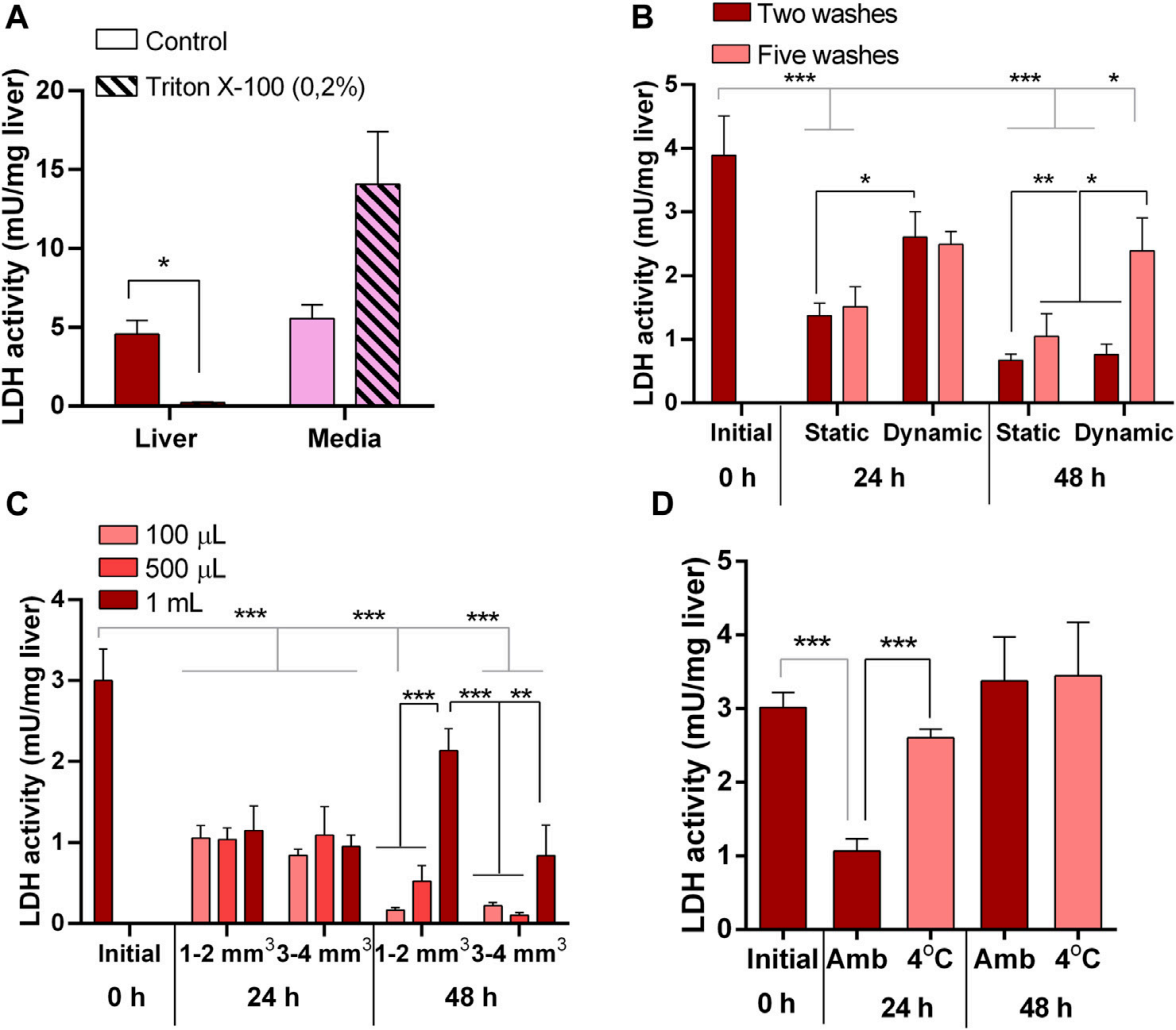
Liver explants culture optimization. **(A)** Lactate dehydrogenase (LDH) activity in liver explants (red) and culture media (pink) as suitable indicator of tissue integrity responding to induced damage by 0.2% Triton X-100. **(B)** LDH activity in liver explants showing the effects on tissue integrity of number of previous washing steps (two and five) and agitation (static and dynamic) during 48 h in culture. **(C)** LDH activity in liver explants showing the effects on tissue integrity of volume of culture media (100 μL, 500 μL, 1 mL) and size of explant (1–2 mm^3^ and 3–4 mm^3^) during 48 h in culture. **(D)** LDH activity in liver explants showing the effects on tissue integrity of temperature (ambient, 4^o^C) during tissue processing prior culture for 48 h.

**FIGURE 3:**
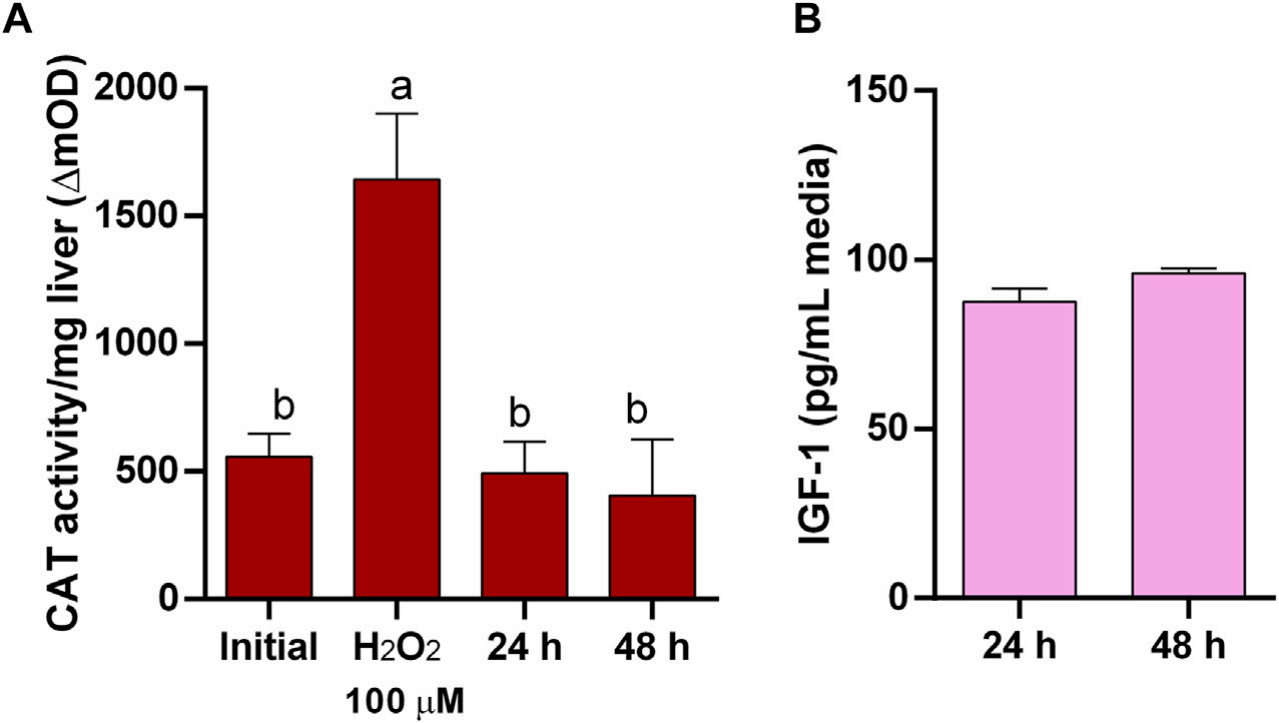
Liver explants functionality. **(A)** Catalase (CAT) activity in liver explants as suitable indicator of oxidative stress responding to induced oxidative stress by H_2_O_2_ and showing low level of oxidative stress in culture. **(B)** Insulin-like growth factor-I (IGF-I) secretion by liver explants in culture.

We first evaluated the LDH activity as a tissue integrity indicator under our assay conditions by inducing damage in explants with a non-ionic surfactant. A 24 h exposure of pituitary (Figure 1A) and liver (Figure 2A) explants to 0.2% Triton X-100 reduced LDH activity in the tissues and increased it in the media, indicating that LDH release is a sensitive indicator of tissue damage in the explants. Also, to ensure that CAT activity can be used as a reliable indicator of oxidative stress under our conditions, both tissues were exposed to H_2_O_2_ and were able to respond increasing its catalase activity (Figure 1C; Figure 3A for pituitary and liver, respectively).

Conversely, in untreated pituitaries, LDH in the media did not increase, glands retained its LDH content (Figure 1B), and maintained the same CAT activity (Figure 1C) and continued secreting GH into the media (Figure 1D) during 48 h. On the other hand, initial attempts to maintain liver explant failed, with more than 50% of LDH recovered in the media after 24 h. Thus, we went through a series of optimization steps, and we followed the progression by means of tissue LDH as recommended for tissues that do not growth in culture and may exhibit spontaneous release of the enzyme (Cox et al., 2021). This approach also allowed comparisons between explants and liver samples taken directly from the animals. LDH retained in the tissue was higher under agitation after 24 h (Figure 2B). The effect of washing septs was more evident at 48 h, with liver explants washed five times retaining the highest LDH activity (Figure 2B), although in all cases it was lower than in the original tissue (Figure 2B). Further improvement in tissue integrity was achieved by decreasing the size of liver explants down to 1–2 mm^3^ and increasing the volume of media to 1 mL (Figure 2C). Finally, by processing the tissue at 4^o^C, the content of LDH in the explants was as in the original tissue after 48 h of culture (Figure 2D). Under these conditions, liver explants showed no signs of oxidative stress (Figure 3A), and kept stable the secretion of IGF-I into the media (Figure 3B).

**FIGURE 4:**
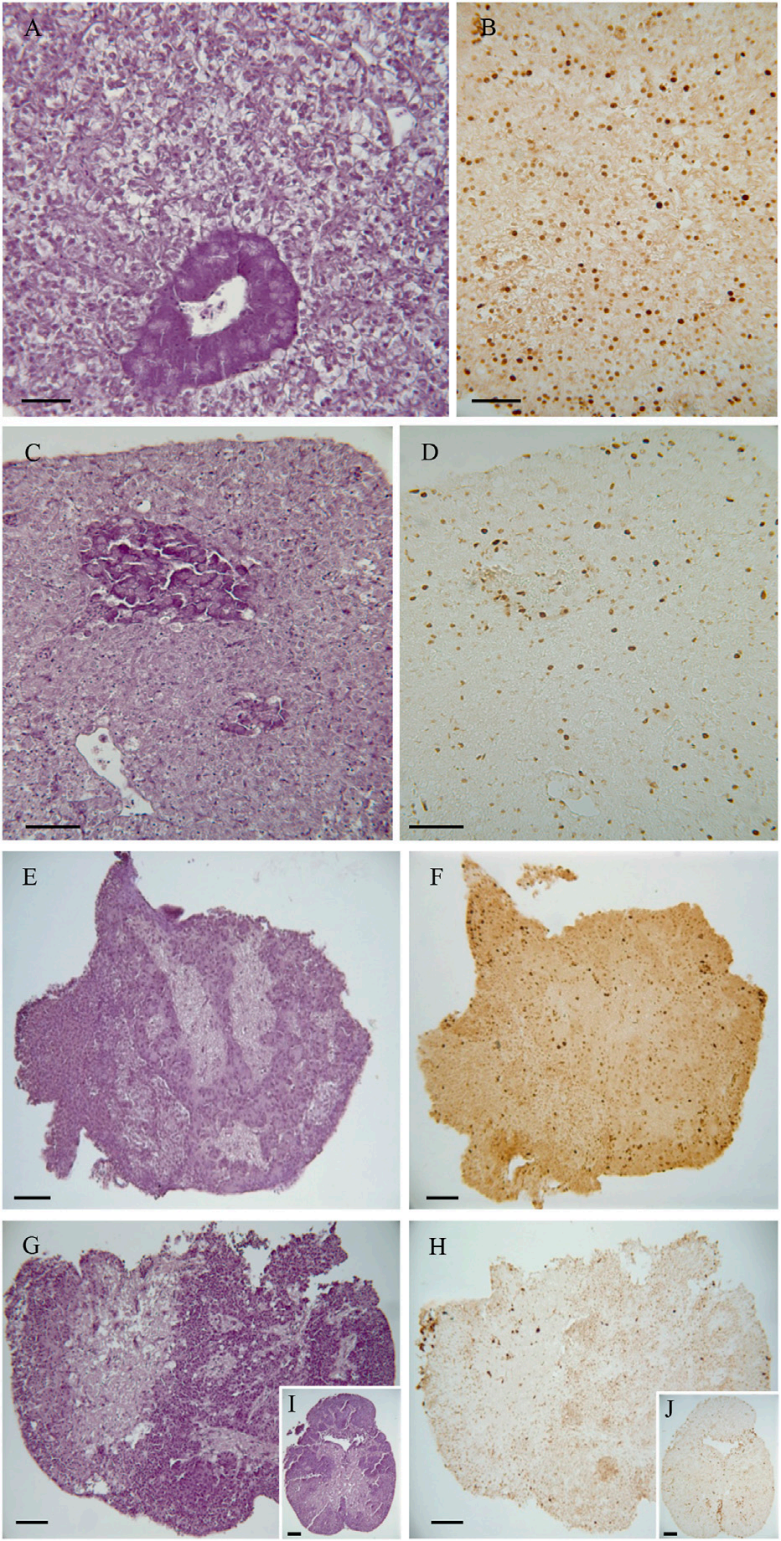
Representative images of the histological analysis of native tissue (directly from fish) and 48 h cultured explants. **(A,B)** Liver native explants. **(C,D)** Liver explants at 48 h. **(E, F)** Pituitary native explants. **(G–J)** Pituitary explants at 48 h. Staining: **(A**, **C**, **E**, **G**, and **I)**, Haematoxylin and Eosin; **(B,D,F,H,J)**, PCNA immunostaining. Scale bar = 50 µm.

Histological evaluation of hepatic and pituitary explants revealed that they also maintained their normal architecture and cellular organization during the 48-h culture period. Figure 4 displays representative images of the explants from all the groups. Overall, the hepatic tissue maintained its structural integrity and cellular characteristics during the 48-h culture period (Figures 4A–D). At time 0, the hepatic tissue sections exhibited a normal parenchymal architecture. The hepatocytes appeared polygonal in shape and were arranged in cords, with a thickness of 2 cells, situated among the sinusoids. Interestingly, pancreatic acinar cells were also observed within the hepatic tissue. After 48 h of culture, the parenchymal arrangement of the liver remained preserved. The hepatocytes maintained their roundish polygonal cell body, and their nuclei were clearly visible, typically containing one nucleolus. However, in the exocrine pancreas, there was a discrete disorganization of the pancreocytes, although they still retained their zymogen granules (Figure 4A–D). Likewise, the histological evaluation of the pituitary tissue showed no significant changes or alterations in its structure during the 48-h culture period. At time 0, the pituitary tissue exhibited two main regions: the adenohypophysis composed of glandular tissue and the neurohypophysis composed of nervous tissue containing nerve fibers from the hypothalamus. After 48 h of culture, the pituitary tissue maintained its standard structural characteristics. Both the adenohypophysis and neurohypophysis remained intact, and the cell types present in these regions retained their characteristic features (Figure 4E–J).

### Explants performed DNA synthesis and cell proliferation for 48 h

We utilized Proliferating cell nuclear antigen (PCNA) immunostaining to investigate the presence of actively dividing cells in the hepatic and pituitary explants. PCNA is a protein that plays a crucial role in DNA replication and repair, and its expression is indicative of cells in the active phase of the cell cycle, specifically during DNA synthesis (S phase). After 48 h of culture, positive PCNA cells were detected in both the hepatic and pituitary explants, indicating that DNA synthesis and cell proliferation were still occurring at this time point. The presence of positive PCNA cells after 48 h of culture indicates that the explanted hepatic and pituitary tissues remained viable and capable of cellular division during the experimental period (Figure 4B,D,F,H,I).

### Key genes of GH-IGF-I axis in liver were modulated *in vitro* by GEN and DAC

The exposure of pituitaries to the assayed concentrations of SAM, GEN and DAC produced no changes in *gh* gene expression (Supplementary Table S2).

**FIGURE 5:**
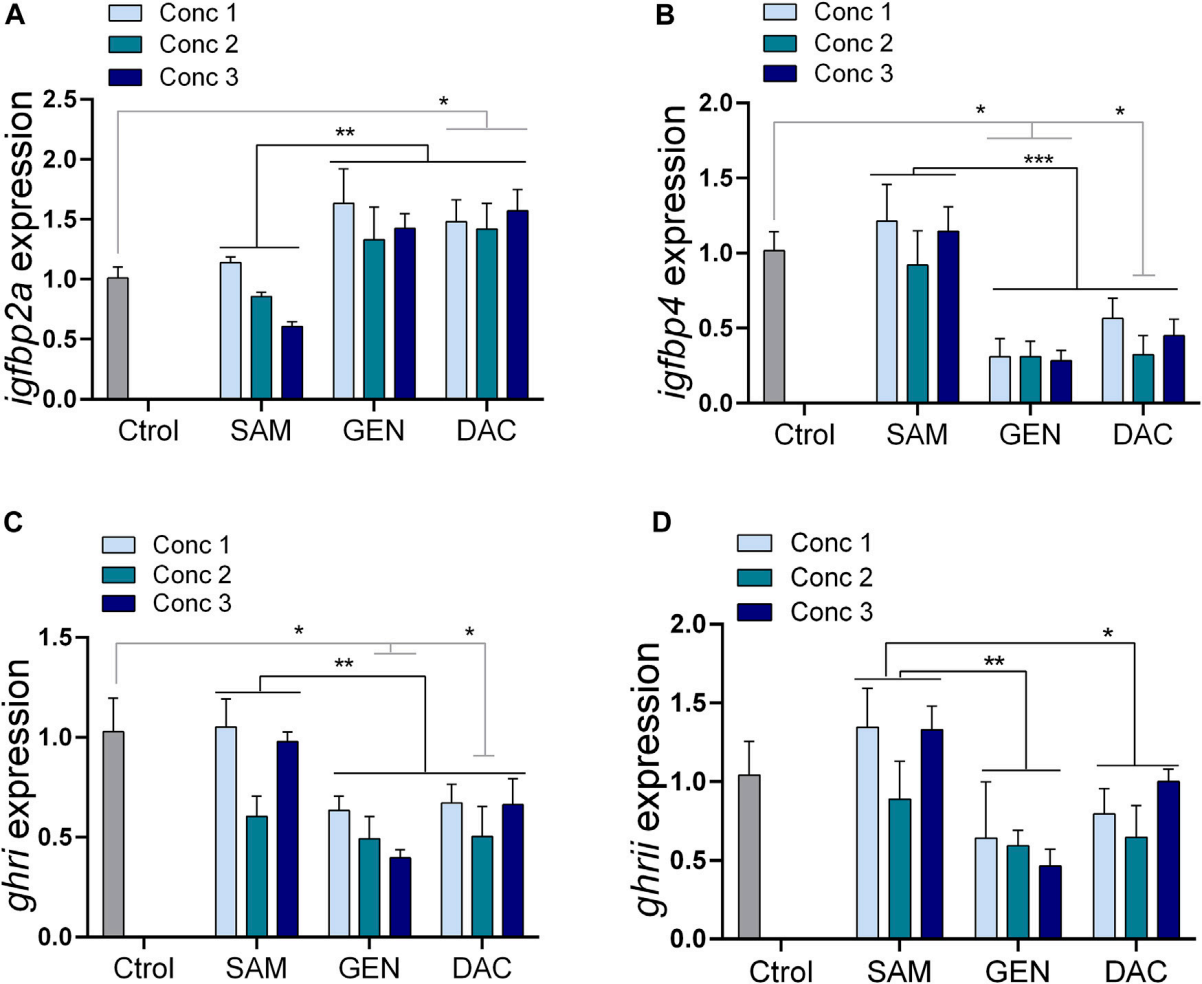
Effects of DNA methylation remodeling factors [S-adenosylmethionine (SAM), genistein (GEN) and 5-Aza-2′-deoxycytidine (DAC)] on the expression of growth-related genes in liver explants. **(A)** *igfbp2a*, **(B)** *igfbp4*, **(C)** *ghri*, **(D)** *ghrii*. Concentrations denoted as 1, 2 and 3 because different for each substance: SAM (100, 200, 300 µM), GEN (5, 10, 20 µM), and DAC (5, 15, 20 µM). Treatments compared with Two-way ANOVA (*p* < 0.05) followed by the Turkey test, and all treatments against the control with one-way ANOVA (*p* < 0.05) followed by a Dunnett multiple comparison test. *p < 0.05, **p < 0.01.

The exposure of liver explants to SAM for 24 h did not produce variations in the expression of the genes studied regardless of the SAM concentration used (Supplementary Table S2). Likewise, the exposure of liver explants to DAC, at the concentrations tested, did not alter the expression of *igf1*, *igfbp1a*, *igfbp1b*, and *igfbp2b* (Supplementary Table S2). However, DAC produced a significant increase in the expression of *igfpb2a*, and a significant decrease in the expression of *igfpb4*, *ghri*, and *ghrii*, respect to SAM treated explants and control livers taken directly from the animal (Table 1; Figure 5). These effects were consistently observed irrespective of the DAC concentration (Figure 5). The effects of GEN were, in general, similar to those of DAC (Figure 5). No interaction was found between the DNA methylation remodeling agents evaluated (SAM, GEN, DAC) and their concentration for the affected (Table 1) and unaffected (Supplementary Table S2) genes.

**TABLE 1:** Statistics for gene expression in liver explants after 24 h exposure to DNA methylation remodeling agents.

Gene	F	P	Significance
*igfbp2a*			
Remodeling agent	8.678	0.0017	**
Concentration	1.244	0.3076	ns
Interaction	1.785	0.1679	ns
All vs. control	2.551	0.0338	*
*igfbp4*			
Remodeling agent	18.87	<0.0001	***
Concentration	0.9075	0.4188	ns
Interaction	0.2524	0.9050	ns
All vs. control	5.998	0.0002	***
*ghri*			
Remodeling agent	6.850	0.0051	**
Concentration	3.258	0.0585	ns
Interaction	0.9620	0.4488	ns
All vs. control	3.708	0.0053	**
*ghrii*			
Remodeling agent	6.628	0.0059	**
Concentration	1.128	0.3427	ns
Interaction	0.6780	0.6148	ns
All vs. control	2.394	0.0440	*

SAM, GEN, and DAC assayed at different concentrations. Results of Two-way ANOVAs, with significance level set at 0.05 are shown. ns: not significant differences, *p < 0.05, **p < 0.01, ***p < 0.0001. Additionally, to compare all conditions against the control, one-way ANOVA, followed by a Dunnett multiple comparison test was used. Statistics for all other genes analyzed but unresponsive to treatments are shown in Additional file 1: Supplementary Table S2.

### DAC produced tissue-specific differences in the expression of DNA methylation related genes

**FIGURE 6:**
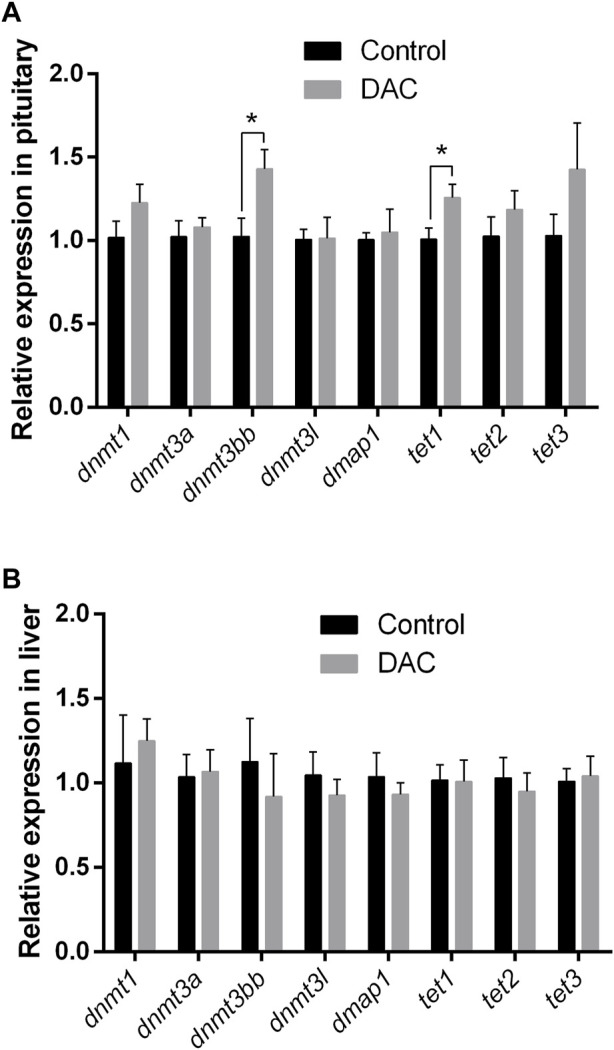
Effects of 24 h exposure of explants to 5-Aza-2′-deoxycytidine (DAC) on the expression of DNA methylation remodeling related genes (*dnmt1*, *dnmt3a*, *dnmt3bb*, *dnmt3l*, *dmap1*, *tet1*, *tet2*, *tet3*). **(A)** pituitary. **(B)** liver explants Control *versus* treated explants compared by t-test (*p* < 0.05). *p < 0.05.

The expression of different genes related with DNA methylation and de-methylation was examined after 24 h exposure of explants to 15 µM DAC. We observed a significant increase in the expression of *dnmt3bb* and *tet1* genes in pituitary (Figure 6A). While no variations were observed in the pituitary expression of other DNA methylation related genes (i.e., *dnmt1*, *dnmt3a*, *dnmt3l* and *dmap1*), a non-significative trend was noticed for *dnmt1, tet2* and *tet3*, with increments in their expression values after DAC exposure (Figure 6A). Conversely, DAC produced no variations in the expression of the methylation-related genes analyzed in liver explants (Figure 6B).

### Activity of DNMT and TET enzymes in explants after DAC exposure agreed with the expression pattern observed

**FIGURE 7:**
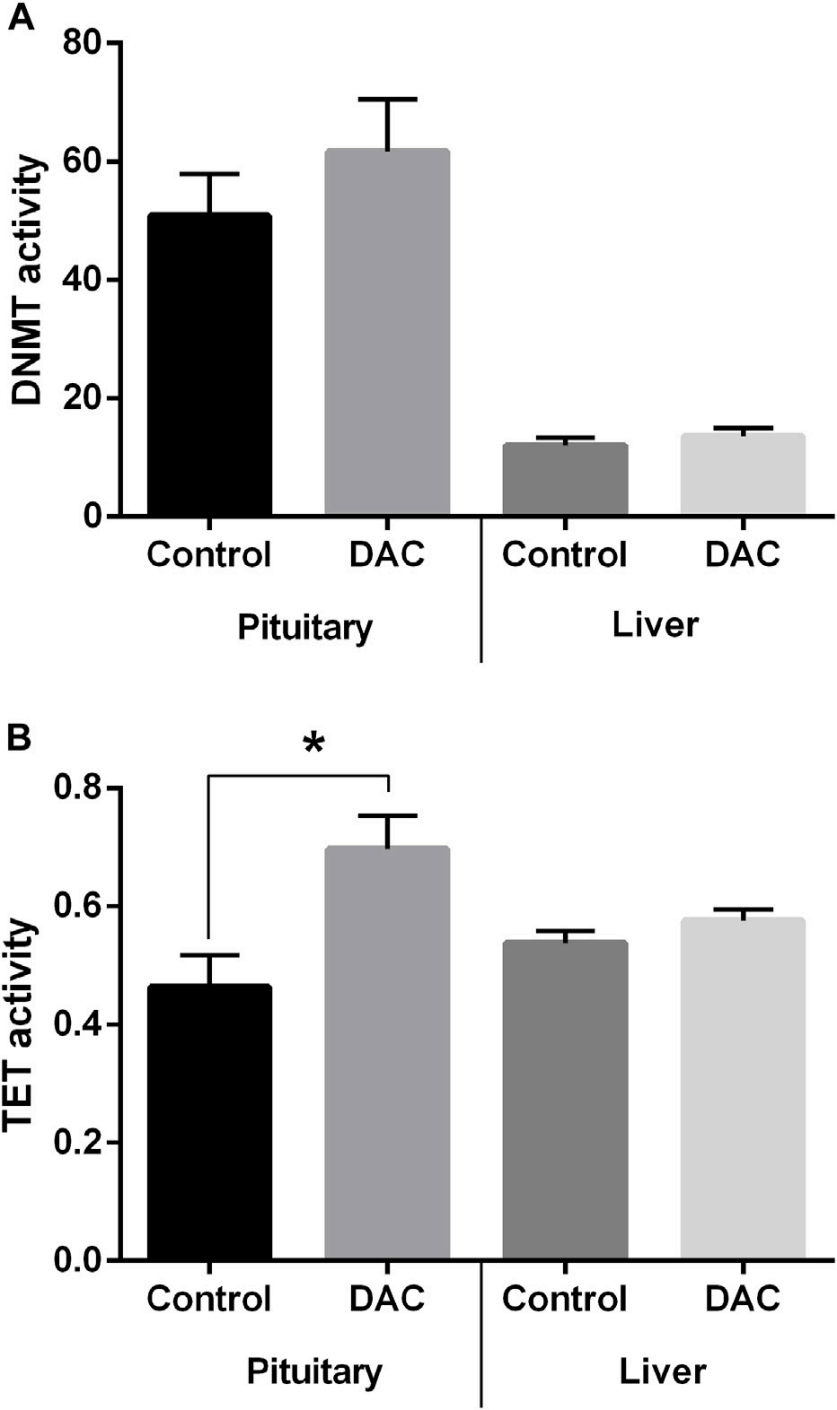
Effects of 24 h exposure of pituitary and liver explants to 5-Aza-2′-deoxycytidine (DAC) on the activity of DNA methylation remodeling enzymes. **(A)** DNA methyltransferase (DNMT) activity. **(B)** Ten-eleven translocation (TET) methylcytosine dioxygenases activity. Control *versus* treated explants compared by t-test (*p* < 0.05). *p < 0.05.

DNMT activity in pituitary and liver exhibited no significant variations due to DAC, but a trend for DNMT activity to be higher in treated pituitaries (Figure 7A) was observed. However, TET activity did increase after DAC exposure in pituitary (Figure 7A), while remained the same in liver explants (Figure 7B).

### Overall DNA methylation and hydroxymethylation in explants after DAC exposure

**FIGURE 8:**
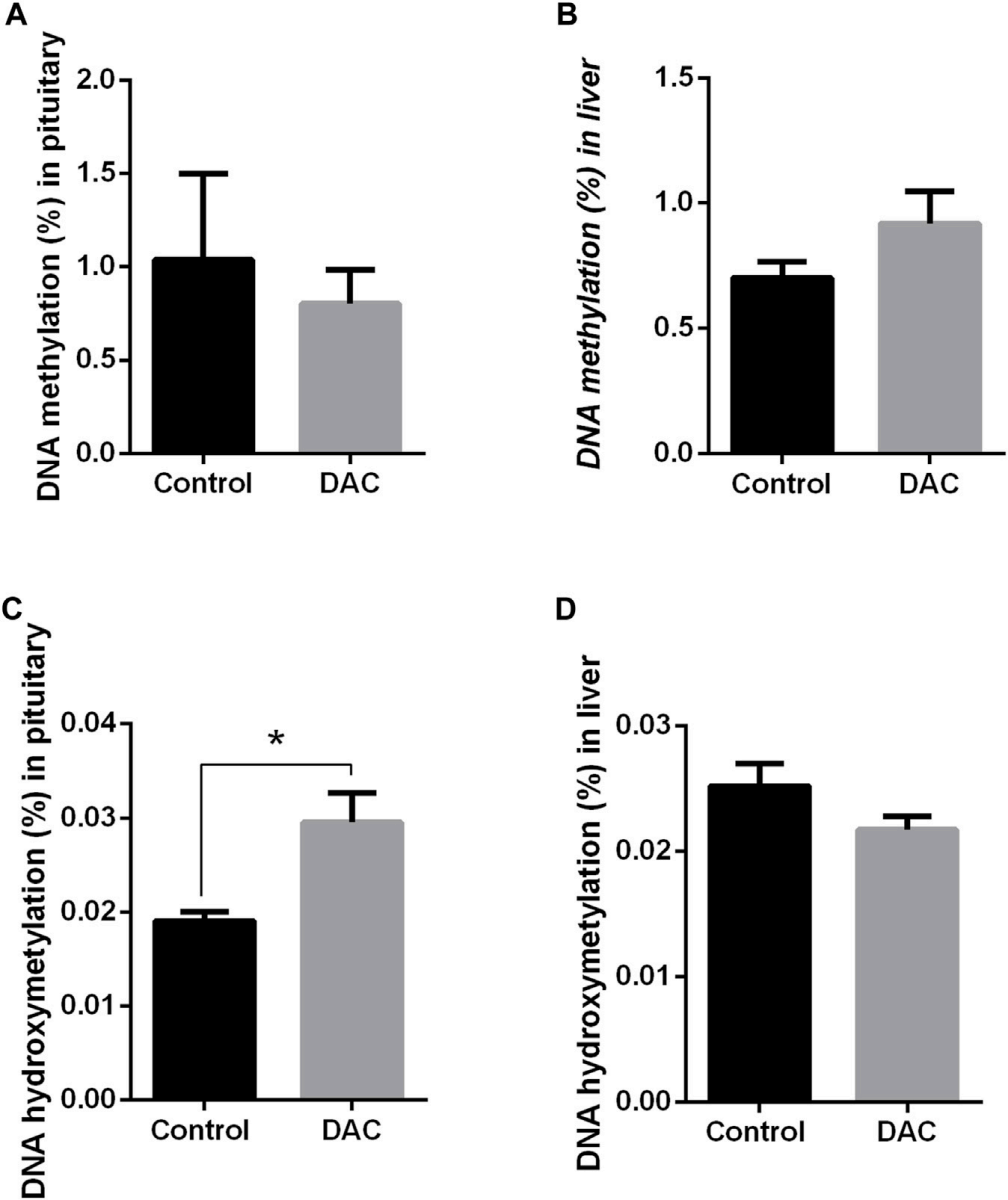
Effects of 24 h exposure of pituitary and liver explants to 5-Aza-2′-deoxycytidine (DAC) on DNA methylation (%5 mC) and in hydroxymethylation (%5 hm C). **(A)** pituitary, 5 mC. **(B)** liver explants, 5 mC. **(C)** pituitary, 5 hm C. **(D)** liver explants, 5 hm C. Control *versus* treated explants compared by t-test (*p* < 0.05). *p < 0.05.

DAC produced opposing effects on DNA methylation and hydroxymethylation within each tissue (Figure 8), though statistical differences were found only for hydroxymethylation in pituitary, which increased due to DAC treatment (Figure 8C).

## Discussion

Although temperature, photoperiod, salinity, pollutants, stocking density, among other factors, affect GH and IGF-I expression and growth rate in fish (Triantaphyllopoulos et al., 2020), the main environmental factor regulating their GH/IGF-I axis is the nutritional status (Pérez-Sánchez et al., 2018; Bertucci et al., 2019). Nutrition may affect DNA methylation by altering the availability of methyl donors (e.g., SAM), by changing the activity of enzymes responsible for the production of SAM, or by activating or inhibiting DNMTs and TETs enzymes (Kadayifci et al., 2018; Skjærven et al., 2022). There are now several lines of evidence indicating that DNA methylation/hydroxymethylation are involved in the modulation of GH/IGF-I axis in fish (Li et al., 2017; Huang et al., 2018; Podgorniak et al., 2019; Konstantinidis et al., 2021). While nutritional interventions targeting the epigenome are a new avenue to improve growth and other phenotypes in farmed fish (Gavery and Roberts, 2017), little is known in most species. In this work, we evaluated the value of tissue explants as tools for broadening our understanding of GH/IGF-I axis regulation in a farmed fish, the gilthead seabream, and to study the effects of epigenetic modulators on this axis.

### Tissue explants retain integrity, functionality, and proliferation

We demonstrated that fish pituitaries and liver explants are viable and functional during 48 h. This experimental window is equal or longer than that used in 78% of the research published in the last decade with PCLS (Dewyse et al., 2021), while explants are cheaper and easier to implement than PCLS. Also, despite the long history of explant culture in fish, many articles never indicate why specific culture conditions were chosen and others provide incomplete descriptions of explant health (LeClair, 2021). Here, we described in detail the optimization process for pituitary and, in particular, liver explants, which resulted problematic in first attempts. LDH is a cytosolic enzyme released into the cell culture medium upon plasma membrane leakage and it has been widely used to assess viability in cultured cells and explants (Cox et al., 2021). Catalase, on the other hand, is one of the most important antioxidant enzymes and it is a reliable marker of oxidative stress (Kocalar et al., 2023). The value of both biomarkers in both tissues was demonstrated before their use during explant optimization. We proved that under our conditions, the cytoplasmic enzyme LDH is retained in the tissue, and that explants are able to respond to induced oxidative stress by increasing CAT activity, hence low CAT activity in untreated explants indicated low level of oxidative stress during culture. Also, we showed that explants were able to express and secrete hormones (GH in pituitary and IGF-I in liver explants) which make them suited to study GH-IGF-I axis.

As histological examination of organ culture is essential to ensure a healthy tissue (LeClair, 2021), our observations were complemented by histology, which revealed that both explants retain their structural integrity and organization. Moreover, we showed that cell proliferation occurred in the explants as in the live fish. This represents additional evidence of viability, being also relevant in the context of epigenetic studies as it may determine the balance between the two modes of action of DNA methylation inhibitors such as DAC. DAC needs cell proliferation to be passively incorporated into DNA and block further DNA methylation by acting as a suicide substrate for DNMT, but can also decrease methylation in the absence of cell division by replication-independent DNMT depletion (Pleyer and Grei, 2015). In this sense, the absence or excess of cell proliferation, which do not occur in our explants, may lead to *in vitro* result that cannot be used for *in vivo* extrapolation.

### GH-IGF-I axis in liver is modulated *in vitro* by DAC

By exposing explants to SAM, GEN and DAC, we explored the possibility to modulate GH-IGF-I axis genes through interventions targeting the epigenome, by affecting overall DNA methylation potential or inhibiting DNMTs. SAM is the main methyl donor in cells, GEN is a plant-derived phytoestrogen that decreases DNMT activity probably by interacting with the catalytic domain of the enzymes (Xie et al., 2014), and DAC is a demethylating agent targeting more strongly DNMT1, although also affecting DNMT3s (Pleyer and Grei, 2015).

Overall, GEN and DAC produced similar effects on gene expression in this study, but we acknowledge that the mechanisms are likely not the same, and in the case of GEN, not necessarily linked with changes in DNA methylation. Although both are inhibitors of DNA methylation, genistein modulates the expression of DNMTs and other chromatin modifiers such as histone deacetylases (HDAC), inhibits the enzymatic activity of DNMTs, HDACs, and histone methyltransferase (HMTs), and can also bind to estrogen receptors activating different pathways (Sundaram et al., 2019). In contrast, DAC targets directly the DNA and binds to the catalytic binding domain of DNMTs (Pleyer and Grei, 2015). Thus, at least for DAC, the observed effects on gene expression are likely to be caused mainly through altering DNA methylation. We found that GEN and DAC increased the expression of *igfbp2a*, and decreased the expression of *igfbp4* and both GH receptors. In rainbow trout (*Oncorhynchus mykiss*), the intraperitoneal injection of GEN also reduced expression of both GH receptors (Cleveland and Manor, 2015). However, in that study, no differences were found for *igfbp4* and *igfbp2a*, which were responsive in our *in vitro* assays in gilthead seabream. Changes in expression of all *igfbp2a, igfbp4, ghri* and *ghrii* after DAC treatment suggest that these genes are modulated, directly or indirectly, by DNA methylation in seabream. Other studies have found evidences of this regulation for *igfbp2* in fish (Podgorniak et al., 2019), mice (Kammel et al., 2016) and humans (Boughanem et al., 2021), for *igfbp4* in sheep (Tian et al., 2022) and humans (Diaz et al., 2020) and for *ghrs* in fish (Zhao et al., 2015) and broilers (Cong et al., 2023). Finally, while changes in DNA methylation at the *igf1* gene of some fish were reported (Li et al., 2017; Huang et al., 2018), we found no evidence of such regulation in the gilthead seabream as judged by the lack of response of this gene to DAC. However, it is worth mentioning that in this study, total *igf1* was measured (i.e., primers annealing to all three forms; Tiago et al., 2008), and the effects on the three *igf1* should be further assessed. IGF-Ic is the most highly expressed form in the liver and it is thought to play systemic roles, while the other transcripts may have a local action (Tiago et al., 2008).

It is difficult to predict the consequences for the fish of the expression pattern produced *in vitro* by DAC. In higher vertebrates, IGFBP2 is believed to bind both IGF-I and IGF-II, modulates IGFs systemic actions, but also acts locally preventing adipogenesis (Boughanem et al., 2021). However, *igfbp2b*, which comprise more that 70% of *igfbp* mRNAs in the gilthead sea bream (Pérez-Sánchez et al., 2018) was unaffected in our study, and the role of *igfbp2a* paralog, which was upregulated by GEN and DAC, is poorly understood though suspected to have also growth inhibitory activity. Yet, *igfbp4* is also abundant in this species, accounting for more than 20% of *igfbp* mRNAs (Pérez-Sánchez et al., 2018), and was downregulated by GEN and DAC in this study. This *igfbp* has growth promoting effects in some fish (Garcia de la Serrana and Macqueen, 2018) thus its reduction might compromise normal growth. Also, in this line, the other two genes downregulated by GEN and DAC were GH receptors, which are crucial for initiating GH signal transduction pathways (Pérez-Sánchez et al., 2018). Besides, because of the functional diversification of *ghr* in fish, both GEN and DAC may affect different physiological processes in the fish, as *ghri* has been more clearly related with growth while *ghrii* is more related with stress response (Saera-Vila et al., 2007; 2009; Pérez-Sánchez et al., 2018). As the gene expression pattern obtained after treatments with DNA methylation inhibitors is suggestive of impaired growth, it is proposed that a proper methylation environment within the liver may ensure growth in this fish species as shown in salmon (Saito et al., 2021; Adam et al., 2022). An *in vivo* grow out trial is underway to clarify this issue, which would have practical implications in feed design for this fish species.

### DAC produced different DNA methylation remodeling conditions in pituitary and liver explants

Nutritional interventions such as the inclusion of DNA methylation inhibitors or enhancers imply that all tissues become exposed, though at different concentrations, to the agents. Thus, it is important to establish if they have synergist or overriding effects on different tissues. SAM supplementation of culture media is known to surprisingly causes both increase and decrease in DNA methylation in different liver cell lines (Wang et al., 2017). We did not address this issue because no effects of SAM exposure on the expression of GH-IGF-I axis genes analyzed were found in liver and pituitary explants. However, this does not mean that SAM had no effect on DNA methylation of explants, which is worthy to be investigated in further studies. Direct evidence of the impact of DNA methylation changes, if occurs, on a gene expression is often difficult to obtain. Indeed, it has been shown in salmon that one-carbon metabolism nutrients affected DNA methylation profiles of genes involved in various cellular processes without directly changing their gene expression in the liver (Saito et al., 2021), and a similar lack of clear relationship was observed for different growth-related genes in large yellow croaker (*Larimichthys crocea*) (Zhang et al., 2019). Also, it should be considered that in addition to DNA, SAM can transfer its methyl group to a large variety of acceptor substrates, including RNA and proteins (Cederbaum, 2010), and other nutrients may be more effective to produce an increase in the methylation potential of tissues (Kharbanda et al., 2005; Saito et al., 2021). Liver explants described here would be useful for screening the potential of those nutrients to produce changes in DNA methylation.

Conversely, here it is showed that DAC produces different effects on liver and pituitary DNA methylation remodeling. While no variations were observed in DAC-treated liver explants in the expression of the DNA methylation remodeling genes studied, in DNMT or TET activities, and accordingly, nor in overall 5 mC and 5 h m, pituitary explant were more responsive. DAC increased the expression of *dnmt3bb* and *tet1* in pituitary, producing increments in both DNMT and TET activity, though more pronounced for TET. Likewise, in other models (i.e., colorectal cancer cells), DAC increased *DNMT3A* and *TET*s gene expression (Gerecke et al., 2018). A non-statistically significant increase in *dnmt1* was observed in pituitary due to DAC exposure. In mouse this gene contains a methylation-sensitive DNA element, which after being demethylated by 5-azadeoxycytidine, results in the induction of *dnmt1* expression (Slack et al., 1999), although opposed results have been obtained in colon cancer cells (Gerecke et al., 2018). Despite DAC increased both hypermethylation and demethylation potential in fish pituitary as reported before in mice (Colwell et al., 2021), the overall result in our assay was only an increase in DNA hydroxymethylation (5 hm C), likely due to a more pronounced increase in TET activity. These enzymes regulate active turnover of DNA methylation by oxidizing 5 mC iteratively to yield first 5 hm C and then two other intermediates (5-formylcytosine, 5 fC; 5-carboxycytosine, 5caC) that can be excised and return to unmethylated cytosines by thymine-DNA glycosylase (TDG)-mediated base excision repair (Zhang et al., 2023). However, full demethylation is not always accomplished, and there are strong evidences supporting that 5 hm C is also a stable epigenetic mark, and not only a demethylation intermediate (Zhang et al., 2023). Indeed, it has been proposed that cytosine hydroxymethylation may contribute to the phenotypic plasticity of growth through epigenetic regulation of the somatotropic axis in Nile tilapia (Konstantinidis et al., 2021). Thus, pituitary explant cultures reported here proved to be useful to gain more insights on this regulation in fish. In addition, it is worth mentioning that site-specific *de novo* hypermethylation due to the action of *dnmt3bb* would be physiologically relevant in pituitary and deserves further examination.

Collectively, our results demonstrate that exposure to a common DNA methylation remodeling agent produces tissue-specific responses in their endogenous DNA methylation machinery, and this may in turn determine the effects in each tissue. Regarding the gilthead seabream tissues examined, pituitary seems to respond stronger to DAC and to have more broad effects on DNA methylation/hydroxymethylation than liver. Yet, although less evident at the overall level, changes in liver seems to be functionally relevant as DAC had clear effects on the expression of some genes from the IGF-I axis. This study highlights the need for genome-wide single-base resolution approaches to truly assess the processes modulated through epigenetics and their regulatory mechanisms in both tissues, for which tissue explants stand as a suitable option, also in line with the principles of the 3Rs (Replacement, Reduction and Refinement).

## Conclusion

This study demonstrates that tissue explants of liver and pituitary are useful for studying epigenetic mechanisms and GH/IGF-I axis regulation in fish. Explants can be used in further studies for the screening of nutrients, additives, and environmental conditions with putative epigenetic effects on important physiological processes performed by pituitary and liver. Evidences provided in this study suggest that DNA methylation or hydroxymethylation may be involved in the regulation of some genes of the GH/IGF-I axis in gilthead seabream. However, it must be argued that we did not attempt to establish a direct connection between DNA methylation and gene expression with our experiment or results. This relationship is hard to disentangle by target-specific approaches without previous information on the genomic regions affected. For instance, changes in DNA methylation several kilobases away from a gene may impact its expression, have different effects depending on the genomic feature affected, or may have occurred not in the studied genes but in other genes upstream in their regulation cascade. An ongoing study at our laboratory is addressing this issue by genome-wide single-base resolution approaches, using herein described pituitary and liver explants as experimental models.

## Data Availability

The raw data supporting the conclusion of this article will be made available by the authors, without undue reservation.

## Abbreviations

18S rRNA 18S ribosomal RNA; 5-hmC 5-hydroxymethylcitocine; 5-mC 5-methylcytocine; ACTB β actin; CAT catalase activity; DAC 5-Aza-2′-deoxycytidine; DMAP1 DNA Methyltransferase 1 Associated Protein 1; DNMT DNA Methyltransferase; EF1A elongation factor 1 α; GAPDH glyceraldehyde-3-phosphate dehydrogenase; GEN genistein; GH growth hormone; GHR GH receptor; GHREs growth hormone response elements; IGFBPs insulin-like growth factor binding proteins; IGF-I insulin-like growth factor I; LDH lactate dehydrogenase; PCNA proliferating cell nuclear antigen; PMS post-mitochondrial supernatant; RPL27A ribosomal protein L27; RPS18 ribosomal protein S18; SAM S-adenosylmethionine; TET tet methylcytosine dioxygenase.
