# Supplementary material for: Tissue explants as tools for studying the epigenetic modulation of the GH-IGF-I axis in farmed fish
Source: Front Physiol. 2024 Jun 20;15:1410660. doi: 10.3389/fphys.2024.1410660 (PMC11222784; doi:10.3389/fphys.2024.1410660)
Supplement: Supplementary file 1 [file Table1.docx]

Supplementary Material

# Supplementary Tables

**Supplementary Table S1.** Genes and qPCR primers (5’- 3’) used in this study. HKG: housekeeping genes.

| **Organ** | **Gene** | **Primer Sequences (5–3)** | **Accession Number /Gene ID** | **References** |
| --- | --- | --- | --- | --- |
| **Pituitary** | *gh* | F: CGTCTCTTCTCAGCCGAT  R: GCTGGTCCTCCGTCTGC | U01301.1 | Mohammed-Geba et al., 2015 |
|  |  |  |  |  |
| **Liver** | *ghr1* | F: ACCTGTCAGCCACCACATGA  R: TCGTGCAGATCTGGGTCGTA | AF438176 | Calduch-Giner et al., 2003 |
|  | *ghr2* | F: GAGTGAACCCGGCCTGACAG  R: CGGTGGTATCTGATTCATGGT | AY573601 | Saera-Vila et al., 2005 |
|  | *igfbp1a* | F: AGTGCGAGTCCTCTCTGGAT  R: TCTCTTTAAGGGCACTCGGC | KM522771 | Vélez et al., 2016 |
|  | *igfbp1b* | F: GCCAAACAGTGTGAGTCATC  R: ATCTTCTTCCCGTTCCAGG | MH577189 | Naya-Català et al., 2021 |
|  | *igfbp2a* | F: CCAGCAAAGAGACCACCT  R: TCTTCATCTCCTGCCTGTG | MH577190 | Naya-Català et al., 2021 |
|  | *igfbp2b* | F: AGCGATGTGTCCTGAGATAGTGAG  R: GCACCGTGGCGTGTAGACC | AF377998 | Naya-Català et al., 2021 |
|  | *igfbp4* | F: GGCATCAAACACCCGCACAC  R: ATCCACGCACCAGCACTTCC | KM658998 | Naya-Català et al., 2021 |
|  | *igf1 (abc)** | F: ACAGAATGTAGGGACGGAGCGAATGGAC  R: TTCGGACCATTGTTAGCCTCCTCTCTG | EF688016 | Tiago et al., 2018 |
|  |  |  |  |  |
| **HKG** | *actb* | F: TCTTCCAGCCATCCTTCCTCG  R: TGTTGGCATACAGGTCCTTACGG | X89920 | Martos-Sitcha et al., 2013 |
|  | *ef1a* | F: AGAGGCTGTCCCTGGTGA  R: TGATGACCTGAGCGTTGAAG | AF184170 | Aedo et al., 2019 |
|  | *18S rRNA* | F: AACCAGACAAATCGCTCCAC  R: CCTGCGGCTTAATTTGACTC | AY993930 | Martos-Sitcha et al., 2015 |
|  | *gapdh2* | F: CATGAAGCCAGCAGAGATCC  R: GGTGGCCGGGTCATATTTC | FM145063 | Salmerón et al., 2016 |
|  | *rps18* | F: AGGGTGTTGGCAGACGTTAC  R: CTTCTGCCTGTTGAGGAACC | AM490061 | Riera-Heredia et al., 2019 |
|  | *rpl27a* | F: ACAACTCACTGCCCCACCAT  R: CTTGCCTTTGCCCAGAACTT | AY188520 | Domínguez et al., 2017 |
|  |  |  |  |  |
| **Pituitary and Liver** | **DNA-methyltransferases** | | | |
|  | *dnmt1* | F: CCTGATTGTGGCAAGTGTGC  R: TTAACTGCCAGGTTTGGGCA | 115575267 | This work |
|  | *dnmt3l* | F: CGGAGCACCTACGGGTTAC  R: CTCTGCTGCCACTGGAGTAT | 115574210 | This work |
|  | *dnmt3a* | F: CCATTCGTGTCCTGTCGCTA  R: GAGTCTTCGCACACTTCCGA | 115597760 | This work |
|  | *dnmt3ba* | F: GCTGAAGCTGATGCTTGACTG  R: CAGACTCCGAGGACTCTTGC | 115585308 | This work |
|  | *dnmt3bb* | F: GGGACTCAAACCTACGCACA  R: GCTGGGGAAATACTCTGGCA | 115585491 | This work |
|  | *dmap1* | F: GACGAGCCGAAAAGAGGGTA  R: CAGTGTGACCCCTGCTGATT | 115590937 | This work |
|  | **Ten-Eleven Translocated enzymes** | |  |  |
|  | *tet1* | F: CTCCCAGGGGTGTCCAATAG  R: GCCAGGATGAGGATCACCAA | 115596529 | This work |
|  | *tet2* | F: GTACACAAGGGTGCCCCATA  R: TACGTTCCCGCACTAACACC | 115579074 | This work |
|  | *tet3* | F: AAGCAAAATGCCACGCAAGT  R: GCCACCTCAGTTGCCAGATT | 115582422 | This work |

*For total *igf1* expression, comprising splice variants a, b and c.

**Supplementary Table S2.** Statistics for gene expression in pituitaries and liver explants after 24h exposure to DNA methylation remodeling agents (SAM, GEN, DAC) at different concentrations. Results of Two-way ANOVAs with significance level set at 0.05 are shown. ns: not significant differences, * P<0.05, ** P<0.01, *** P< 0.0001. To compare all conditions against the control (DMSO), One-way ANOVA followed by a Dunnett multiple comparison test was used.

| **PITUITARY** | | | |
| --- | --- | --- | --- |
| ***gh: Two-way ANOVA*** | | | |
| Remodeling agent | F = 0,898 | P = 0,422 | ns |
| Concentration | F = 0,108 | P = 0,745 | ns |
| Interaction | F = 0,114 | P = 0,893 | ns |
| ***gh: One-way ANOVA*** | | | |
| All *vs* control | F = 0.257 | P = 0.782 | ns |
| **LIVER** | | | |
| ***igf1: Two-way ANOVA*** | | | |
| Remodeling agent | F = 2.624 | P = 0.095 | ns |
| Concentration | F = 2.779 | P = 0.083 | ns |
| Interaction | F = 0.387 | P = 0.815 | ns |
| ***igf1: One-way ANOVA*** | | | |
| All *vs* control | F=1.599 | P=0.1718 | ns |
| ***igfbp1a: Two-way ANOVA*** | | | |
| Remodeling agent | F = 2.043 | P = 0.153 | ns |
| Concentration | F = 1.736 | P = 0.1995 | ns |
| Interaction | F = 1.477 | P = 0.243 | ns |
| ***igfbp1a: One-way ANOVA*** | | | |
| All *vs* control | F= 1.746 | P=0.1327 | ns |
| ***igfbp1b: Two-way ANOVA*** | | | |
| Remodeling agent | \|  \|  \| \| --- \| --- \|   F = 1.316 | P = 0.2894 | ns |
| Concentration | F = 1.471 | P = 0.2523 | ns |
| Interaction | F = 0.646 | P = 0.6355 | ns |
| ***igfbp1b: One-way ANOVA*** | | | |
| All *vs* control | F= 0.9573 | P= 0.4980 | ns |
| ***igfbp2a: Two-way ANOVA*** | | | |
| Remodeling agent | F = 8.678 | P = 0.0017 | ** |
| Concentration | F = 1.244 | P = 0.3076 | ns |
| Interaction | F = 1.785 | P = 0.1679 | ns |
| ***igfbp2a: One-way ANOVA*** | | | |
| All *vs* control | F= 2.551 | P= 0.0338 | * |
| ***igfbp2b: Two-way ANOVA*** | | | |
| Remodeling agent | F = 3.419 | P = 0.0509 | ns |
| Concentration | F = 0.3512 | P = 0.7077 | ns |
| Interaction | F = 0.2858 | P = 0.8840 | ns |
| ***igfbp2b: One-way ANOVA*** | | | |
| All *vs* control | F=1.089 | P= 0.4063 | ns |
| ***igfbp4: Two-way ANOVA*** | | | |
| Remodeling agent | F = 18.87 | P < 0.0001 | *** |
| Concentration | F =0.9075 | P = 0.4188 | ns |
| Interaction | F = 0.2524 | P = 0.9050 | ns |
| ***igfpb4: One-way ANOVA*** | | | |
| All *vs* control | F=5.998 | P= 0.0002 | *** |
| ***ghri: Two-way ANOVA*** | | | |
| Remodeling agent | F= 6.850 | P= 0.0051 | ** |
| Concentration | F= 3.258 | P= 0.0585 | ns |
| Interaction | F= 0.9620 | P= 0.4488 | ns |
| ***ghri: One-way ANOVA*** | | | |
| All *vs* control | F= 3.708 | P=0.0053 | ** |
| ***ghrii: Two-way ANOVA*** | | | |
| Remodeling agent | F= 6.628 | P=0.0059 | ** |
| Concentration | F= 1.128 | P= 0.3427 | ns |
| Interaction | F= 0.6780 | P= 0.6148 | ns |
| ***ghrii: One-way ANOVA*** | | | |
| All *vs* control | F=2.394 | P=0.0440 | * |

**References**

Aedo JE, Ruiz-Jarabo I, Martínez-Rodríguez G, Boltaña S, Molina A, Valdés JA, et al. Contribution of Non-canonical Cortisol Actions in the Early Modulation of Glucose Metabolism of Gilthead Sea Bream (*Sparus aurata*). Front Endocrinol (Lausanne). 2019;10:779. <https://doi.org/10.3389/fendo.2019.00779>

Calduch-Giner JA, Mingarro M, Vega-Rubín de Celis S, Boujard D, Pérez-Sánchez J. Molecular cloning and characterization of gilthead sea bream (*Sparus aurata*) growth hormone receptor (GHR). Assessment of alternative splicing. Comp Biochem Physiol Part B Biochem Mol Biol. 2003;136:1-13. <https://doi.org/10.1016/s1096-4959(03)00150-7>

Domínguez D, Rimoldi S, Robaina LE, Torrecillas S, Terova G, Zamorano MJ, et al. Inorganic, organic, and encapsulated minerals in vegetable meal based diets for *Sparus aurata* (Linnaeus, 1758). PeerJ. 2017;5:e3710. <https://doi.org/10.7717/peerj.3710>

Martos-Sitcha JA, Campinho MA, Mancera JM, Martínez-Rodríguez G, Fuentes J. Vasotocin and isotocin regulate aquaporin 1 function in the sea bream. J Exp Biol. 2015;218:684-693. <https://doi.org/10.1242/jeb.114546>

Martos-Sitcha JA, Wunderink YS, Gozdowska M, Kulczykowska E, Mancera JM, Martínez-Rodríguez G. Vasotocinergic and isotocinergic systems in the gilthead sea bream (*Sparus aurata*): an osmoregulatory story. Comp Biochem Physiol A Mol Integr Physiol. 2013;166(4):571-81. <https://doi.org/10.1016/j.cbpa.2013.09.001>

Mohammed-Geba K; Mancera, JM; Martínez-Rodríguez G. Acclimation to different environmental salinities induces molecular endocrine changes in the GH/IGF-I axis of juvenile gilthead sea bream (*Sparus aurata L.*). J Comp Physiol B Bioch Syst Envir Physi. 2015;185(1):87-101. <https://doi.org/10.1007/s00360-014-0871-7>

Naya-Català F, Simó-Mirabet P, Calduch-Giner J, et al. Transcriptomic profiling of Gh/Igf system reveals a prompted tissue-specific differentiation and novel hypoxia responsive genes in gilthead sea bream. Sci Rep. 2021;11:16466. <https://doi.org/10.1038/s41598-021-95408-6>

Riera-Heredia N, Lutfi E, Gutiérrez J, Navarro I, Capilla E. Fatty acids from fish or vegetable oils promote the adipogenic fate of mesenchymal stem cells derived from gilthead sea bream bone potentially through different pathways. PLoS One. 2019;14(4):e0215926. <https://doi.org/10.1371/journal.pone.0215926>

Saera-Vila A, Calduch-Giner JA, Pérez-Sánchez J. Duplication of growth hormone receptor (GHR) in fish genome: gene organization and transcriptional regulation of GHR type I and II in gilthead sea bream (*Sparus aurata*). Gen Comp Endocrinol. 2005;142: 193–203. <https://doi.org/10.1016/j.ygcen.2004.11.005>

Salmerón C, Riera-Heredia N, Gutiérrez J, Navarro I, Capilla E. Adipogenic ggene expression in gilthead sea bream mesenchymal stem cells from different origin. Front Endocrinol (Lausanne). 2016;7:113. <https://doi.org/10.3389/fendo.2016.00113>

Tiago DM, Laizé V, Cancela ML. Alternatively spliced transcripts of *Sparus aurata* insulin-like growth factor 1 are differentially expressed in adult tissues and during early development. Gen Comp Endocrinol. 2008;157:107–115. <https://doi.org/10.1016/j.ygcen.2008.04.006>

Vargas-Chacoff L, Astola A, Arjona FJ, Martín del Río MP, García-Cózar F, Mancera JM, et al. Gene and protein expression for prolactin, growth hormone and somatolactin in *Sparus aurata*: seasonal variations. Comp Biochem Physiol B Biochem Mol Biol. 2009;153(1):130-5. <https://doi.org/10.1016/j.cbpb.2009.02.008>

Vélez EJ, Sheida A,  Millán-Cubillo A,  Fernández-Borràs J, Blasco J,  Jin Chan S, et al. Effects of sustained exercise on GH-IGFs axis in gilthead sea bream (*Sparus aurata*). Am J Physiol Regul Integr Comp Physiol. 2016;310:R313-R322. <https://doi.org/10.1152/ajpregu.00230.2015>
